# A Complex Intervention to Strengthen Person-Centered Care and Leadership in Residential Care Facilities (the PERLE Study): Protocol for a Development and Implementation Study

**DOI:** 10.2196/76185

**Published:** 2025-07-21

**Authors:** Anna-Karin Edberg, Annica Backman

**Affiliations:** 1 Faculty of Health Sciences Kristianstad University Kristianstad Sweden; 2 Department of Nursing Faculty of Medicine Umeå University Umeå Sweden

**Keywords:** person-centered care, leadership, residential care facilities, nursing homes, long-term–care facilities, older people, quasi-experimental intervention

## Abstract

**Background:**

Although the benefits of person-centered care (PCC) are widely recognized, there is a lack of empirical research on how PCC is best developed and implemented by the leaders responsible for driving such initiatives. To achieve meaningful change and ensure the sustainability of PCC practices, it is crucial to understand how leaders can foster care environments rooted in person-centered values while managing operational challenges. This knowledge gap highlights the need for an in-depth exploration of the conceptual foundations, experiences, mechanisms, strategies, and outcomes of person-centered leadership (PCL) to design an intervention for strengthening such leadership.

**Objective:**

The Person-Centered Care and Leadership in Residential Care Facilities (PERLE) study aims to (1) explore and develop tools to measure PCL; (2) develop, test, implement, and investigate the effects of an intervention to strengthen PCL in residential care facilities (RCFs) for older people; and (3) generate knowledge about the process of development and implementation of the intervention.

**Methods:**

The PERLE study builds on the Medical Research Council framework for the development of complex interventions. It includes several studies with exploratory, descriptive, correlational, and quasi-experimental designs and is based on the research group’s previous research on PCL. The project is composed of 5 work packages (WPs). Each WP includes research questions with different samples; data collection; and methodological approaches, such as qualitative, mixed methods, and quantitative studies. As this project involves sensitive issues, a high level of ethical awareness was maintained throughout. The primary challenge is the possible interference with participants’ work time, which could otherwise be devoted to supporting older people and staff. Nevertheless, the involvement of leaders and staff can lead to valuable knowledge that can improve the quality of PCC. Participants will be provided with both oral and written information about the study and assured of their right to withdraw at any time without providing a reason.

**Results:**

As of July 2025, 6 studies in WP I have been conducted, and one is in progress. The conducted studies address leaders’ understanding of PCC, the meaning of PCL in RCFs from the perspective of leaders, the ethical challenges in providing PCC during the COVID-19 pandemic, and the cultural adaptation of the aged care clinical leadership qualities framework.

**Conclusions:**

This project aims to provide new insights into the support that first-line managers need to advance PCC in RCFs, highlight their specific challenges, and create tailored support measures for the implementation of PCL. The intervention could potentially reduce staff turnover and related costs while supporting leadership training and education to benefit future leaders in aged care, which may be important from an international perspective.

**International Registered Report Identifier (IRRID):**

DERR1-10.2196/76185

## Introduction

### Overview

The need for research on person-centered leadership (PCL) stems from the growing recognition of person-centered practices and their well-documented benefits for patients, staff, and care cultures, particularly in the context of care for older people [1]. Despite the theoretical promise of person-centered care (PCC), leading PCC remains challenging because of structural, cultural, and systemic barriers [2]. Leaders often face conflicting demands between focusing on individual needs and meeting organizational goals [3], which can be ethically challenging when leading PCC. This became particularly visible during the COVID-19 pandemic [4]. Furthermore, while PCC has been extensively explored, there is limited empirical evidence on how PCC can be developed and implemented by the leaders who are appointed to steer such processes. Understanding how leaders can support care environments permeated by person-centered values, while balancing operational pressures, is essential for promoting meaningful changes and sustainable PCC practices. This gap in knowledge calls for a deeper investigation into the conceptual meanings, experiences, mechanisms, strategies, and outcomes of PCL. The knowledge gained will facilitate the development of a complex intervention to strengthen PCL. This study protocol describes the conceptualization and design of the Person-Centered Care and Leadership in Residential Care Facilities (PERLE) study.

### Background

#### PCC Approach

The PERLE study is grounded in person-centeredness, originally based on Rogerian psychotherapy [5]. During the 1990s, Kitwood [6] adapted the ideas for dementia care formulated by Rogers [5] to address “malignant social psychology,” a term he coined to describe practices that devalued, dehumanized, and depersonalized persons with dementia. Kitwood [6] emphasized the importance of recognizing personhood as preserved, even amid cognitive decline. Building on the work by Kitwood [6], Brooker and Latham [7] introduced the VIPS framework for PCC, with the acronym representing 4 core components: valuing human life regardless of cognitive ability, individualized care, the perspective of the user, and a supportive environment. The importance of the relationship between the person and the caregiver is emphasized in the conceptual framework for person-centered practice by McCormack [8]. It advocates a holistic, ethical, and humanistic foundation; shared decision-making; and care based on the person’s needs, values, and preferences, ensuring their well-being remains the priority [9]. PCC also involves promoting quality of life by incorporating meaningful activities, social interactions, and cultural elements [10].

Several intervention studies have provided compelling evidence for the importance of PCC, particularly for older people, establishing it as an indicator of high-quality care in residential facilities, especially for persons living with dementia [11]. A systematic review and meta-analysis of PCC interventions revealed several positive outcomes, including reduction in agitation, neuropsychiatric symptoms, and depression, along with improved quality of life among older people in residential care facilities (RCFs) [12]. These findings call for the reshaping of care for older people, making it more tailored and personalized. Essential components of person-centered interventions include social interaction, physical activities, cognitive training, life story work, emotional support, education and support for caregivers, and adjustments to the care environment and organization [13].

These findings offer an understanding of key elements and activities for improving PCC practices. Furthermore, McCormack and McCance [1,14] stress the importance of a supportive organization, competent staff, and an adaptive care environment. A supportive organization fosters creativity, initiative, and collaboration among staff, underpinned by strong leadership. Ultimately, PCC places the person’s preferences and needs at the forefront, guiding decisions and actions. This also means that leadership focusing on person-centeredness has to address the needs of the persons who receive care and create a supportive care and work environment, while balancing organizational goals with person-centered principles.

#### Leading PCC

Previous research highlights the critical importance of leadership in successfully implementing PCC in RCFs [15]. Leaders play a vital role in shaping a professional vision, acting as role models, and offering support by actively engaging in the implementation process [16]. Stein-Parbury et al [17] emphasize that building trustful relationships through support and active engagement with staff is key to achieving success. Similarly, Chenoweth et al [18] stress that flexibility in work approaches and involving staff in care-related decision-making are pivotal factors in effective implementation. Moreover, Rosemond et al [19] illustrate how leaders can enhance PCC by introducing the approach with responsiveness, inclusivity, and respect. This is reinforced by Jeon et al [20], emphasizing that leadership requires not only listening to staff suggestions but also making necessary adjustments to organizational structures to support person-centered practices. In addition, leadership for PCC practices involves fostering trust through delegation, clarifying roles, considering staff expertise, and creating opportunities for development [21]. Backman et al [22] highlight the leader’s role in supporting staff to provide care tailored to the needs of older persons, while simultaneously focusing on team development and improving workplace cohesion.

Despite the evidence that leadership is important for implementing person-centered interventions, research indicates that practical implementation remains challenging, fragmented, and often not fully person-centered [23]. Cases of unmet goals in person-centered interventions have been attributed to a lack of leadership support to continually motivate, coach, and sustain the process [24] or to management’s failure to enable necessary organizational changes, leading to unfavorable outcomes [18]. These findings underscore the critical role of leadership in initiating and fostering PCC. In a systematic review, Moenke et al [25] found that most studies on leadership for person-centered practices focus on what leaders should do, rather than on how they can be supported in developing their leadership capabilities. This gap between person-centered theory and practice highlights the need for further research to understand how leaders can be supported in promoting PCC daily over the long term.

Insufficient leadership support has also been linked to challenges for staff to address the unique needs of older people [26], reinforcing the need for targeted leadership development in PCC. In addition, research also emphasizes the importance of restoring humanistic aspects in care to enhance quality, improve staff well-being, and increase job satisfaction [3]. It has been said that leaders who heavily rely on hierarchical structures tend to prioritize tasks and outcomes over the humanistic elements of leadership, often neglecting the development of employees’ skills [27]. While leadership is central to PCC, it also introduces new demands and challenges for leaders. The concept of PCL, by contrast, remains largely underexplored, resulting in a significant knowledge gap that needs to be addressed to support leaders in developing their PCL skills.

#### Leading PCC During the COVID-19 Pandemic

The COVID-19 pandemic highlighted the need for leadership that addresses the needs of both staff and older people [28], underscoring the importance of leadership practices aligning with person-centered principles. PCC involves navigating complex ethical and practical challenges, even under ordinary circumstances. These challenges became significantly greater during the COVID-19 pandemic, as the need to protect populations considered vulnerable introduced restrictions and shifts in care practices. As in many other countries, Sweden faced significant challenges, with early efforts focused on protecting older persons [29]. In RCFs, numerous restrictions were implemented [30], limiting the ability to provide PCC. Leaders struggled to adapt their leadership as care conditions kept changing [31], while staff faced new ethical dilemmas, such as when isolating residents by confining them to their rooms against their will [32]. The restrictions severely impacted residents’ autonomy, freedom, participation, and overall well-being [30]. Although the COVID-19 pandemic entailed specific conditions, it also exposed preexisting ethical challenges in delivering PCC and underscored the importance of PCL.

#### Theoretical Framework of PCL

Skivington et al [33] highlight the importance of using theoretical frameworks in the development of complex interventions. One such framework is the aged care clinical leadership qualities framework (ACLQF), which clarifies clinical leadership, outlines several leadership aspects, and can be used to support the implementation of PCC. PCC forms the foundation of the ACLQF, which emphasizes the importance of staff and managers treating all residents with respect by acknowledging and addressing their unique experiences and needs [34]. A cluster randomized controlled trial evaluating a leadership program based on the ACLQF in the care of older people in Australia [35] showed positive outcomes across all primary measures. These included leaders demonstrating greater individual consideration, increased motivation, stronger role modeling, and more recognition and praise to staff.

#### Measuring and Evaluating PCL

As PCL is a relatively new concept, validated methods for measuring PCL have so far been lacking. Several instruments measuring different aspects of PCC [36] have been translated and adapted for the Swedish context, for example, the Person-Centered Care Assessment Tool [37,38], person-centered climate [39,40], and PCC in acute settings for older persons with cognitive decline [41]. Several general leadership instruments exist [42,43], but none specifically measure leadership in the context of care of older people or PCC. To enable the evaluation of the effects of leadership interventions with a focus on PCL, a specific instrument measuring these aspects is needed.

### Rationale for the Study

Although the benefits of PCC are widely recognized, there is a lack of empirical research on how PCC can be developed and implemented by the leaders responsible for driving such initiatives. To achieve meaningful changes and ensure the sustainability of PCC practices, it is crucial to understand how leaders can foster care environments rooted in person-centered values while managing operational challenges. This knowledge gap highlights the need for an in-depth exploration of the conceptual foundations, experiences, mechanisms, strategies, and outcomes of PCL to design an intricate intervention aimed at strengthening such leadership.

### Aims

The PERLE study aims to (1) explore and develop tools to measure PCL; (2) develop, test, implement, and investigate the effects of an intervention to strengthen PCL in RCFs for older people; and (3) generate knowledge about the process of development and implementation of the intervention.

## Methods

### Context

In Sweden, health care and social care are tax-financed and means-tested, as is placement in an RCF or, using international terminology, in a nursing home, skilled nursing facility, or long-term-care facility. In Sweden, the county councils (regions) are responsible for health care services provided at hospitals and in primary care (health care centers). For people aged ≥65 years, the municipalities are responsible for health and social care, although services may be provided by private actors or nonprofit organizations [44]. The chronological age of 65 years, the former retirement age, is still defined in the legislation as the entry age to an RCF. However, since 2020, a pension age adjustment system has been in place in Sweden, with the present suggested retirement age being 67 years, but with the possibility to retire earlier or to work longer if the employer agrees, meaning that 65 years is no longer a relevant definition of “older people.” Using chronological age instead of functional age as an indicator for when someone is “older” has also been questioned. In Sweden, the term “older people” is primarily used for people aged ≥75 years.

Because of the growing aging population, the demand for more RCF places is increasing, while the number of available places in RCFs has decreased over time [45]. In 2024, approximately 10% of people aged ≥80 years, mostly women, resided in RCFs [46]. Today, the mean age for moving to an RCF is 86 years [46], and the mean length of stay is 2 years [47]. However, this varies greatly. A large percentage of residents die shortly after admission [48]. Residents often have major health care needs related to aging and frailty when they move into residential care, and the majority have a cognitive disorder [49].

The largest staff groups in municipal care for older people are direct nursing staff, that is, licensed practical nurses (61%) and nursing assistants (32%), while registered nurses (RNs) account for 7% [50]. The majority are women (82%), 42% are foreign-born, and 49% have only temporary employment (ie, employed by the hour and only when the employer needs additional staff) [51].

RNs work in the daytime and are on call evenings, nights, and weekends. Occupational therapists and physiotherapists are employed by the municipality and work on a consultancy basis. Physicians also work on a consultant basis in RCFs and are employed by the primary health care center. Physicians are responsible for medical assessments, while RNs coordinate the residents’ medical care. First-line managers are, together with the RNs, responsible for the quality of care, and are responsible for staff management, budgeting, administration, and the work environment [46]. First-line managers mainly have a professional background in social work or nursing. RNs are not subordinate to first-line managers, as they are part of a different organizational structure, often together with occupational therapists and physiotherapists.

During the COVID-19 pandemic, there was no strict lockdown in Sweden, but social distancing was applied, which included visitor restrictions in RCFs [29]. The restrictions were in place for periods between March 2020 and March 2022. The COVID-19 vaccination was introduced in RCFs at the beginning of 2021, and by January 2022, 95% of RCF residents had received a vaccination [52].

### Design

The PERLE study includes several studies with exploratory, descriptive, correlational, and quasi-experimental designs and is based on the research group’s previous research on PCL [53]. The project is composed of 5 work packages (WPs) whose aims are as follows:

- WP I explores PCC and PCL in RCFs for older people. The knowledge derived from this WP will be used as a basis for the development of tools to measure PCL (WP II) and the development of the intervention (WP III).
- WP II develops tools to measure intervention effects concerning PCL in RCFs for older people. The knowledge derived from this WP will be used as primary and secondary outcome measures when investigating the effects of the intervention (WP IV).
- WP III develops and tests an intervention to strengthen PCL in RCFs for older people. The knowledge derived from this WP will be used as a basis for the implementation of the intervention (WP IV) and will, together with the results of WP II, be used as a basis for the calculation of sample sizes and for determining the methods of randomization.
- WP IV implements and investigates the effects of an intervention to strengthen PCL in RCFs.
- WP V generates knowledge about the process of development and implementation of the intervention. WPs II to IV will contribute to the results of this WP.

**Figure 1:**
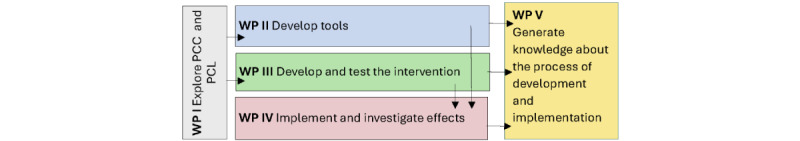
Overview of the different work packages (WPs) and their interrelationships. PCC: person-centered care; PCL: person-centered leadership.

For an overview of the 5 WPs and how they are interrelated, refer to Figure 1.

The first studies will, together with the group’s previous research, be used as a foundation for the development of a complex intervention for leaders in RCFs. Skivington et al [33] highlight that the possibility of success of an intervention increases if it is adjusted to the specific context and builds on an extensive empirical and theoretical basis. Therefore, it is of utmost importance that the various parts of the intervention are developed for the specific context and target group and are based on theoretical underpinnings. This also applies to the evaluation of an intervention, where it is equally important that the outcome measures are developed for the actual context and in relation to the target group.

The guidelines for the development and evaluation of complex interventions have been updated since they were first published in 2008 by the Medical Research Council [54]. The new guidelines [33] describe four phases: (1) development of the intervention (WP I and II), (2) feasibility assessment (WP III), (3) implementation, and (4) evaluation (WP IV). The studies within the PERLE study will mainly generate knowledge to inform the development of the intervention, while also providing insights into the processes of its development, implementation, and evaluation (WP V). Zwarenstein et al [55] point to the importance of involving the users (eg, staff, leaders, and decision makers) in the phases of development, implementation, and evaluation. Most studies and all the WPs in the PERLE study will have a high degree of user involvement.

Skivington et al [33] list six core elements of importance in the intervention development and implementation process:

1. Consider context; contextual factors, such as cultural, social, and economic factors, need to be considered. This is addressed in a process of cocreation with the leaders, especially in WP II and III, ensuring that the intervention is based on the specific context of Swedish residential care.

2. Develop, refine, and test or retest program theory; the program theory should describe how an intervention is expected to lead to effects and under what circumstances. In this project, the program theory is operationalized by a theoretical and empirical evidence base derived from WP I, being developed in WP II, and tested in WP III.

3. Engage stakeholders; stakeholders, in this project being mainly first-line managers, will participate in all stages of the project (WP I-V) in a cocreation process consisting of meetings, workshops, and focus group discussions.

4. Identify key uncertainties; the potential to identify key uncertainties is strengthened by the close involvement of stakeholders and the use of different methods in development and testing (WP II and III).

5. Refine the intervention; the refinement of the intervention components will be done in the feasibility studies in close collaboration with first-line managers (WP III).

6. Identify economic considerations; economic considerations need to be identified and reflected throughout the development and implementation phases to ensure that the intervention is cost-effective. In this project, relevant health economic measures will be developed in WP II, tested in WP III, and used for evaluation in WP IV.

Each WP includes research questions (RQs) with different samples, data collection methods, and methodological approaches. For an overview of the RQs, samples, data collection, and analyses in relation to the different WPs, refer to Table 1.

**Table 1:** Overview of the WPs^a^, RQs^b^, sample, and data collection and analyses.

WPs and RQs		Sample				Data collection					Analyses		
		FLMs^c^	RNs^d^	Direct care staff	Interviews		Questionnaires	Workshops	Literature	Qualitative		Mixed methods	Quantitative
**WP I: explore PCC^e^ and leadership in RCFs^f^ for older people**													
	RQ 1—What are leaders’ understandings of PCC in RCFs?	✓	✓		✓					✓			
	RQ 2—Which ethical challenges emerged in the provision of PCC during the COVID-19 pandemic?	✓	✓	✓	✓					✓			
	RQ 3—What is the meaning of PCL^g^ in RCFs from the perspective of leaders?	✓	✓		✓					✓			
	RQ 4—What is the conceptualization of PCL in RCF from the perspective of RCF leaders?	✓	✓					✓				✓	
	RQ 5—What is the present evidence base of PCL?								✓	✓			
	RQ 6—Is the ACLQF^h^ relevant and applicable in a Swedish RCF context?	✓	✓				✓						✓
**WP II: develop tools to measure intervention effects concerning PCL in RCFs for older people**													
	RQ 7—How can PCL (primary outcome) be measured?	✓		✓			✓						✓
	RQ 8—What other (secondary) outcome measures concerning staff, leaders, and care quality are relevant?								✓				✓
	RQ 9—What health economic outcome measures are relevant, and which economic considerations are needed?								✓				✓
**WP** **III: develop and test an intervention to strengthen** **PCL** **in** **RCFs** **for older people**													
	RQ 10—Which intervention components are relevant and can be adopted in a Swedish aged care context?	✓						✓				✓	
	RQ 11—Are the intervention components feasible, and how are they experienced by the leaders?	✓			✓					✓			
	RQ 12—What are the strengths and challenges of the different parts of the intervention?	✓			✓					✓			
**WP IV: implement and investigate the effects of an intervention to strengthen PCL in RCFs**													
	RQ 13—What are the effects of the intervention?	✓		✓			✓						✓
**WP** **V: generate knowledge about the process of development and implementation of the intervention**													
	RQ 14—How do the participants experience the process of cocreation of the development and implementation of the intervention?	✓			✓		✓			✓			

^a^WP: work package.

^b^RQ: research question.

^c^FLM: first-line manager.

^d^RN: registered nurse.

^e^PCC: person-centered care.

^f^RCF: residential care facility.

^g^PCL: person-centered leadership.

^h^ACLQF: aged care clinical leadership qualities framework.

### Studies With a Qualitative Approach

The qualitative studies concern leaders’ understanding of PCC in RCFs (RQ 1), ethical challenges in the provision of PCC during the COVID-19 pandemic (RQ 2), the meaning of PCL in RCFs from the perspective of leaders (RQ 3), the evidence base of PCL (RQ 5), the feasibility and experience of the intervention components (RQ 11), strengths and challenges of the different parts of the intervention (RQ 12), and the experience of the cocreation process of development and implementation of the intervention (RQ 14).

#### Samples

The sample for the qualitative studies will be purposively selected to include people who can report relevant experiences and describe the phenomenon being investigated [56]. First, permission from the heads of social services representing municipalities of varying sizes in both rural and urban areas in southern, western, and northern Sweden will be collected. Thereafter, first-line managers, RNs, and direct care staff working in RCFs will be invited to participate in the study. They will receive oral and written information about the study at workplace meetings. Interested persons will be asked to contact a member of the research team. The estimated sample size will range between approximately 15 and 20 participants in each group; to ensure variation in experiences, participants will be drawn from different regions of Sweden, diverse workplaces, and will vary in age and work experience [57].

#### Data Collection

Individual and focus group interviews will be performed with leaders and direct care staff. Before data collection, pilot interviews will be conducted to test the interview guide and format. Before the interview, the participants will be asked to provide demographic information. Both the individual and the focus group interviews will be guided by a semistructured interview guide. The focus groups will be relatively small (3-5 participants) to enable digital focus group interviews. Focus group interviews will be conducted by a moderator and an observer. The moderator will facilitate the discussion and ensure that all participants are involved, and the observer will take notes and ask complementary questions [58]. All interviews will be audio recorded, transcribed verbatim, and pseudonymized. The interview guides are in Swedish and are available on request.

For the metasynthesis, data will be extracted from empirical studies retrieved from relevant databases. The review (RQ 5) for establishing the evidence base will be conducted by searches across relevant databases for studies on PCL published in English or a Scandinavian language within the past 20 years, ensuring both relevance and accessibility. Abstracts will be systematically reviewed to assess their alignment with the study’s objectives. Articles that pass this stage will then undergo a quality assessment, following the established guidelines outlined by the Swedish Agency for Health Technology Assessment and Assessment of Social Services [59]. Only studies demonstrating high methodological quality will be included in the analysis.

#### Analyses

The interview material concerning leaders’ understanding of PCC (RQ 1) will be analyzed using a *discourse analytic approach* with a focus on the question “How do leaders talk about PCC?” The analysis will be guided by constructing discourses on PCC to articulate its various aspects and categorize language use regarding specific ways of talking about PCC [60].

The interview material concerning leaders’ experiences of ethical challenges during the COVID-19 pandemic (RQ 2) and the meaning of PCL from the perspective of leaders (RQ 3) will be analyzed using *conventional content analysis,* as described by Hsieh and Shannon [61]. The content analysis will be conducted in several steps: (1) an initial reading of the texts; (2) repeated reading of the transcripts, highlighting text (meaning units) related to the aim; (3) a line-by-line coding of meaning units; (4) grouping of the codes into meaningful clusters; and (5) sorting the clusters by content, grouping content into subcategories, and abstracting to main categories. The analysis will be conducted as a constant iteration between the text, codes, clusters, and possible categories.

The interview material concerning staff experience of ethical challenges in the provision of PCC during the COVID-19 pandemic (RQ 2) and the experience of the cocreation of development and implementation of the intervention (RQ 14) will be analyzed using *thematic analysis*, as described by Braun and Clarke [62]: (1) familiarizing with the data, including multiple readings of the transcripts and a shared discussion of all authors’ initial understanding of the data; (2) coding; (3) sorting the codes into preliminary themes; (4) reviewing and validating the themes in relation to the data; (5) compiling an overview (similar to a thematic map) as a basis for defining and naming the themes; and (6) writing a report [62].

The *metasynthesis* (RQ 5) of the included studies from the database search will be analyzed and synthesized into common themes, covering the content of PCL in existing research. This comprehensive approach will allow for the integration of both theoretical perspectives and empirical evidence. The extracted data will be systematically organized into codes and categories to identify recurring themes, patterns, and variations. Codes and categories will be synthesized into overarching themes that connect the findings to a broader theoretical framework [63]. This will result in a comprehensive compilation of existing evidence regarding PCL.

### Studies With a Mixed Methods Approach

The mixed methods studies will use group concept mapping (GCM) to explore the conceptualization of PCL in RCFs from the perspective of RCF leaders (RQ4) and to refine the intervention components (RQ10).

#### Samples

The sample for the mixed methods studies will likewise be purposively selected to include participants with relevant experience [56]. After permission from the heads of municipal social services has been obtained, RNs (RQ 4) and first-line managers (RQ 4 and RQ 10) will be invited to participate. They will be informed about the study and get in touch with the research team if interested, as is the case with the qualitative studies. The minimum sample size for a GCM study is 20 participants, although a larger sample of 25 to 40 is often used to achieve a more robust result. The sample size for the GCM studies ranges from 30 to 40 [64].

#### Data Collection and Analyses

Data will be collected and analyzed using GCM. This is a collaborative process that combines qualitative and quantitative data collection and analysis with a specific focus. In RQ 4, the characteristics of PCL are the focus, while the focus of RQ 10 is the identification of intervention components. GCM is a structured method for organizing and “mapping” ideas, needs, and preferences [65]. It involves the following steps: (1) brainstorming with participants using a prompt; (2) organizing the responses (where additional aspects from RQ 3 will be added for RQ 10, if missing) and entering them into the program Groupwisdom (Concept Systems Inc); (3) participants rate the responses in relation to how important and how feasible the response is on a 4-point scale where 1=“not at all” and 4=“very much”; (4) analyzing the responses based on their relationships (qualitative analysis) and ranking (basis for quantitative analysis); (5) conducting quantitative analysis of the organization and ranking in the Groupwisdom program, resulting in ≥1 visual figures; and (6) interpreting the visual figures together with the participants [66].

### Studies With a Quantitative Approach

The studies with a quantitative approach involve the translation and cultural adaptation of the ACLQF (RQ 6); development of an instrument to measure PCL (RQ 7); identification of other relevant outcome measures concerning staff, leaders, and care quality (RQ 8); and identification of health economic outcome measures and economic considerations (RQ 9). The quantitative studies will have correlational (RQ 6 and 7) and descriptive (RQ 8 and 9) designs.

#### Samples

The sample for the studies with correlational designs will be composed of RNs (RQ 6), first-line managers (RQ 6 and 7), and direct care staff (RQ 7). The estimated sample size for RQ 6 is 30 participants (15 RNs and 15 first-line managers), as the preferred range for calculation of item-level content validity index (I-CVI) is approximately 10 experts from each group [67]. The estimated sample size for RQ 7 is 40 first-line managers participating in the development of the scale and approximately 100 direct care staff for the evaluation of its psychometric properties. The sample size of 100 is preliminary, as it depends on the number of items in the scale and needs to be adequate for the calculation of both internal consistency and test-retest reliability, as well as for exploratory factor analysis [68]. The studies with a descriptive design will be based on literature searches (RQs 8 and 9).

#### Data Collection and Analyses

The framework will initially be translated into Swedish using forward and backward translation (English-Swedish-English) by 2 independent translators. Thereafter, the participants will be asked to complete a web survey in which they will rate the relevance of the 40 translated statements in the framework on a 5-point scale, ranging from 1=“not at all relevant in a Swedish context” to 5=“to a high degree relevant in a Swedish context.” The test of the prefinal version, the cultural validation, will be examined using the I-CVI, where the number of participants giving the rating “relevant” (3 or 4) for each item will be divided by the total number of participants. When all participants rate an item as relevant, the I-CVI is 1.0. As recommended by Polit et al [67], a scale can be judged as having excellent content validity if the items have an I-CVI of ≥0.78. The cultural adaptation of the ACLQF has already been finalized and published (refer to the Results section).

The development of an instrument to measure PCL in RCFs from the perspective of staff, the PCL Questionnaire, will follow the Consensus-Based Standards for the Selection of Health Measurement Instruments (COSMIN) guidelines [69]. The development will be conducted in four phases: (1) deriving items based on the results from RQ 5, (2) preliminary testing, (3) analysis, and (4) psychometric evaluation [68]. The developed instrument will be used as the primary outcome measure when investigating intervention effects.

In the first phase, relevant statements (items) will be developed through the following steps: (1) formulation of specific items, where statements are carefully crafted to be clear, concise, and easy to understand; (2) face validity testing through cognitive interviews with managers and staff to evaluate the comprehensibility and interpretability of the items and to identify potential issues; and (3) revisions based on feedback, where items are revised for clarity and precision according to the feedback obtained during the interviews.

In the second phase, preliminary testing of the instrument will be conducted using classical test theory to identify and minimize measurement errors. Classical test theory will also be used to reduce the number of items and thus optimize the instrument’s efficiency. In the third phase, a 2-parameter item response theory model will be applied to gain a detailed understanding of how individual items function regarding difficulty and discrimination. This model addresses how well each item differentiates between participants with varying degrees of the ability in focus (ie, varying degrees of PCL). In the fourth phase, psychometric evaluations will be conducted to ensure the instrument’s reliability and validity. This fourth step will also include a test-retest assessment to examine the instrument’s stability over time, as well as refinement and adjustments to the instrument following the results of the psychometric evaluations. The instrument will be freely available for research purposes and academic use. Commercial exploitation and use in business activities will not be permitted without prior written approval from the authors.

Relevant outcome measures concerning leaders, staff, care quality, and economic measures (RQs 8 and 9) will be identified through a systematic search of the literature, where instruments showing reliable and valid psychometric properties will be selected. The identified instruments will be discussed in the steering group and the reference group regarding quality, feasibility, and the possibility of detecting intervention effects. These instruments will be used as secondary outcome measures when investigating intervention effects.

### The Intervention

The insights gained from the earlier WPs (WP I-III; Figure 1) will guide the development of the intervention content, structure, and evaluation methods. A cluster randomized quasi-experimental study will be conducted, focusing on PCL through a complex intervention specifically tailored to support first-line managers in the care of older people. This design will ensure that the intervention is evidence based, relevant, practical, and user-friendly, addressing the unique challenges of—and opportunities within—the aged care context. By systematically incorporating knowledge from the earlier phases, the intervention aims to create a well-considered approach to strengthen PCL practices and gradually impact PCC quality. During the initial phases, the program theory will undergo further refinement. The tentative conceptualization posits that by supporting the leaders in practicing PCL, the implementation and overall quality of PCC will increase. This improvement is expected to positively affect the staff’s working environment, working conditions, and work satisfaction, ultimately contributing to the well-being of residents.

As the intervention concerns leaders, an appropriate cluster would be the unit for which each leader is responsible. However, additional information derived from WP III is needed to understand how different units are connected, to estimate the risk of contamination and determine which units should be included in the randomization.

The measurement of intervention effects (RQ 13) will be based on the results from RQs 6 to 9, while the sample will be composed of first-line managers and direct care staff. The sample size will be determined by power analyses based on expected differences in primary and secondary outcome measures.

### Timeline

**Figure 2:**
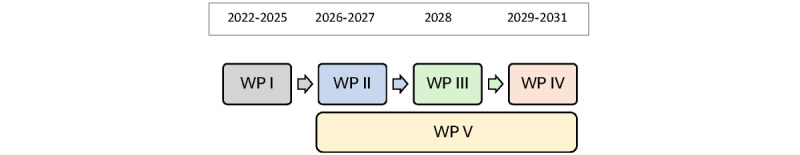
Timeline for the work packages (WPs).

Some studies in WP I started already in 2022 and will be concluded by 2026. WP II will run between 2026 and 2027, WP III will run through 2028, and WP IV is planned to start in 2029. Finally, WP V will run in parallel with WP III and IV (Figure 2).

### Research Group

The research group is multidisciplinary and represents the subjects of nursing, social work, medicine, geriatrics, rehabilitation, and health economics from 4 different universities: Kristianstad University, Umeå University, and Karolinska Institute in Sweden and the University of Sydney in Australia. The group consists of 4 professors, 1 associate professor, 3 researchers, and 2 doctoral students. Additional postdoctoral and doctoral students will be assigned. The University of Sydney will be involved in WP I only, particularly in relation to the adaptation of the framework, while the remaining WPs will be conducted in Sweden.

### Project Organization

The project has a steering group composed of representatives from the municipalities at the government level, the principal investigators, and the coordinators of the 5 WPs, who are responsible for strategic planning and decisions. The project also includes a reference group of direct care staff responsible for ensuring the clinical relevance of the results and the intervention. These groups will meet approximately 4 times a year. The project group will meet every month via a digital meeting forum.

### Ethical Considerations

This project involves several sensitive issues that need to be addressed, and we will promote ethical awareness throughout the project. The main ethical challenge concerns the risk of taking up the participants’ working time, which would otherwise be dedicated to supporting older people in their care and, in the case of the leaders, the personnel. This may be balanced by the fact that the leaders’ participation in the study can contribute to new knowledge, which, in turn, can help improve the quality of care.

In all empirical studies, the participants will receive both oral and written information about the study as well as about their right to withdraw from ongoing participation at any time without needing to state a reason. The participants will report their interest in participating directly to the researchers, thus reducing the risk of pressure from colleagues or managers. All participants will provide written informed consent. Information about the study participants will be coded, with the code list being stored in a safe locker, apart from the data. Only the responsible researchers will have access to the code list. The material will be held in secure custody at Kristianstad University (WP I) and Umeå University B (WP II-V) in accordance with the universities’ regulations concerning data storage and security. The confidentiality of the participants will be kept in the publications by using pseudonymns. All studies will be performed in line with the Declaration of Helsinki. The studies in WP I have been approved by the Swedish Ethical Review Authority (number 2021-000413). Additional ethics approval applications for WPs III-V will be submitted. The participants will not receive any compensation for their participation.

## Results

As of July 2025, a total of 6 studies in WP I have been conducted (relating to RQs 1-3 and RQ 6). Of the 6 studies, 5 are published [70-74] and 1 has been submitted for publication (M Jönsson, unpublished, July 2025). The study related to RQ 4 is in progress.

### Leaders’ Understanding of PCC

The findings indicate that the leaders recognized the importance of considering older people’s autonomy, individual needs, and care environment in PCC. However, their narratives also revealed paradoxes concerning respect for autonomy, the significance of the past and the present, and the shared environment. Leaders play a vital role in emphasizing autonomy while offering practical support to staff navigating these challenges. Achieving PCC involves a delicate balance between meeting residents’ needs, respecting their home environment, and ensuring staff well-being. These findings have recently been corroborated (M Jönsson, unpublished, July 2025).

### The Meaning of PCL in RCFs From the Perspective of Leaders

Our findings indicate that providing PCL comprises 2 elements: being person-centered by focusing on relationships with employees and leading PCC by prioritizing the older persons [70]. The findings highlight that in a complex and fragmented aged care organization, PCL requires not only skills and abilities but also an embodiment of person-centered principles to convey them to employees and older people simultaneously. PCL is defined by leaders’ beliefs, abilities, and actions and not necessarily by their position of authority [71].

### Ethical Challenges in Providing PCC During the COVID-19 Pandemic

The findings show that during the COVID-19 pandemic, leaders needed to prioritize the needs and safety of the society and the care facility over individual residents’ needs, which impacted the possibility of providing PCC. Moreover, it was difficult to balance protecting the residents from infection while preserving their dignity and autonomy [72]. Among the ethical challenges experienced, staff witnessed older persons being deprioritized and receiving unequal care, which violated their human rights. Staff felt constrained and undervalued, with a deep sense of being abandoned. Their own integrity and autonomy were also severely compromised. Fulfilling their professional duty came at great personal risk, as staff had their lives on the line while working in an atmosphere of pervasive fear [73].

### Translation and Cultural Adaptation of the ACLQF

The ACLQF was translated into Swedish, cross-culturally adapted, and validated. It was shown to be useful and suitable for leaders in residential care in Sweden. The framework clarifies the leader’s role and identifies leadership attributes and requirements for PCL in residential care, thereby providing support to leaders by framing PCL [74].

## Discussion

### Anticipated Findings

The PERLE study seeks to achieve an in-depth exploration of the conceptual foundations, experiences, mechanisms, strategies, and outcomes of PCL; develop tools for implementation of a quasi-experimental complex intervention; develop, test, and implement the intervention; and investigate its effects. A further aim is to generate knowledge about the process of development and implementation. So far, several explorative studies have been conducted in WP I; once all studies in WP I are complete, their findings will serve as a foundation for the subsequent WPs.

In WP I, we used a broad definition of “leaders in RCFs,” including both RNs, in their role as clinical leaders, and first-line managers as RCF leaders. However, our findings, along with previous research, for example, by Josefsson and Hansson [75], suggest that Swedish RNs in municipal care for older people do not inherently view themselves as leaders per se. This fact requires attention. In the upcoming studies within the PERLE study, our primary focus will therefore be on first-line managers’ leadership. Our plan is for subsequent studies to focus solely on RNs and their leadership role in the provision of PCC.

### Rigor and Methodological Aspects

There are several methodological aspects to be considered in the outline of the project concerning both the qualitative and quantitative studies. *Qualitative research* is evaluated based on trustworthiness, including credibility, dependability, confirmability, and transferability [76]. One aspect of credibility is accuracy in the selection of participants. In the studies, we will strive for a broad variation in experiences by including direct care staff and leaders representing a range of experience, education, and care contexts. This will also increase the transferability of the findings. Credibility concerns whether the study’s findings are correct and accurate [76]. This is the responsibility of the researchers and depends on the research methods used. Credibility will be strengthened by using well-known and established methods, researcher triangulation, and user involvement. The steering group and reference group will further enhance referential adequacy. Dependability refers to the consistency and reliability of a study’s results and the possibility for other researchers to replicate the study. Therefore, we will provide a thorough description of the context, sample, data collection, and analysis in each study. The confirmability of a study concerns objectivity, accuracy of the material, relevance, and meaning [76]. We will carefully provide audit trails of each step of the data analysis and present and discuss our preunderstandings of the phenomena in focus. Participant quotes will be provided to support the findings. The entire research group will be involved in the synthesis of data, thereby enabling a multifaceted analysis while also reducing the impact of individual researchers’ preunderstanding of the phenomenon. Transferability concerns whether, and to what extent, the results are applicable in other contexts, circumstances, and settings [76]. Therefore, we will provide detailed descriptions of the context, participants, and data collection procedures.

*Quantitative research* is evaluated based on reliability and internal and external validity. Reliability in WP IV will concern the instruments developed or used to investigate intervention effects regarding homogeneity, stability, and equivalence. Each instrument will be critically reviewed for its psychometric properties in line with the COSMIN guidelines [69]. Internal validity embraces aspects of content and face validity, addressing whether the instruments measure what they are intended to measure. In the PERLE study, these aspects will mainly be highlighted in WP II, which has a high degree of user involvement. In WP II, validity—addressing whether a test accurately measures the concept being studied—will be assessed regarding homogeneity, convergence, and theory evidence (construct validity), as well as convergent, divergent, and predictive validity (criterion validity) [56].

The development of the intervention study calls for further attention concerning internal validity. The main potential threats to internal validity will be organizational and economic changes that might affect the outcomes, testing and instrumentation effects as the participants become familiar with the outcome measures, and selection bias in the way the intervention and control units are chosen. As there is a high turnover rate among Swedish RCF staff, aspects of attrition must be considered when calculating the sample size [56]. As leaders and staff in residential care will be closely involved in WP III, there is strong potential to identify risks, especially resistance related to organizational culture, and explore ways to mitigate them.

### Plan for Dissemination

The plan for communicating the results involves different target groups: (1) staff and leaders in the care of older people; (2) decision makers at municipal, regional, and national levels; and (3) the scientific community and society at large, as outlined in Textbox 1.

**Strategies for disseminating the research findings.**

1. The dissemination of the research results for staff and leaders will take place via established collaborations and forums, such as research breakfasts and seminars.
2. The dissemination of research results to decision makers at municipal and regional levels will be achieved via county-wide management seminars and established channels, such as the project steering group.
3. The scientific community and society at large will be informed via several channels, including the following:
  Publications in reputable scientific journals to ensure credibility and reach a broad target group, preferably national and international researchers, as well as relevant education at undergraduate, advanced, and doctoral levels
  National and international conferences, seminars, and workshops where both the research results and the methods and cocreative design will be communicated to, and discussed with, health and care managers in health and care, health care personnel, decision makers in the care of older people, relatives, interest organizations, and international researchers
  Popular science articles in Swedish to reach key target groups in health care and care of older people, as well as audiences outside academia
  Course literature, via already established editorial positions and chapter authorship for teaching materials at undergraduate and advanced levels. The knowledge generated by the project can be included in future editions and thus form the basis for training future care and nursing leaders.

### Conclusions

Ultimately, this project aims to enhance PCC by focusing on the needs and preferences of older people and to improve their quality of life. The PERLE study addresses a key challenge highlighted during the COVID-19 pandemic: balancing the well-being of staff with the care needs of older people. The project aims to provide new insights into the need to support first-line managers in advancing PCC in RCFs, understanding their specific challenges, and creating tailored support measures for the implementation of PCL. Improving PCL is expected to enhance working conditions and address recruitment, retention, and absenteeism challenges in RCFs. The intervention could potentially reduce staff turnover and related costs while supporting leadership training and education to benefit future leaders in aged care. The results will likely be applicable in an international context, that is, beyond Scandinavian countries, especially in countries with similar health care systems. All instruments will be made available for translation and cultural adaptation for research purposes.

## Abbreviations

ACLQF: aged care clinical leadership qualities framework
COSMIN: Consensus-Based Standards for the Selection of Health Measurement Instruments
GCM: group concept mapping
I-CVI: item-level content validity index
PCC: person-centered care
PCL: person-centered leadership
PERLE: Person-Centered Care and Leadership in Residential Care Facilities
RCF: residential care facility
RN: registered nurse
RQ: research question
WP: work package

## Data Availability

The datasets generated or analyzed during this study will available in Swedish from the corresponding author on reasonable request.
